# New insights into automatic treatment planning for cancer radiotherapy using explainable artificial intelligence

**DOI:** 10.1088/1361-6560/ae2561

**Published:** 2025-12-15

**Authors:** Md Mainul Abrar, Xun Jia, Yujie Chi

**Affiliations:** 1 Department of Physics, The University of Texas at Arlington, Arlington, TX, United States of America; 2 Department of Radiation Oncology and Molecular Radiation Sciences, Johns Hopkins University, Baltimore, MD, United States of America

**Keywords:** automatic treatment planning, deep reinforcement learning, explainable artificial intelligence, actor critic with experience replay, integrated gradient method

## Abstract

*Objective.* This study aims to uncover the opaque decision-making process of an artificial intelligence (AI) agent for automatic treatment planning. *Approach.* We examined a previously developed AI agent based on the actor-critic with experience replay (ACER) network, which automatically tunes treatment planning parameters (TPPs) for inverse planning in prostate cancer intensity modulated radiotherapy. We selected multiple checkpoint ACER agents from different stages of training and applied an explainable AI method to analyze the attribution from dose-volume histogram (DVH) inputs to TPP-tuning decisions. We then assessed each agent’s planning efficacy and efficiency, and evaluated their policy space and final TPP tuning space. Combining findings from these approaches, we systematically examined how ACER agents generated high-quality treatment plans in response to different DVH inputs. *Main results.* Attribution analysis revealed that ACER agents progressively learned to identify dose-violation regions from DVH inputs and promote appropriate TPP-tuning actions to mitigate them. Organ-wise similarities between DVH attributions and dose-violation reductions ranged from 0.25 to 0.5 across tested agents. While all agents achieved comparably high final planning scores, their planning efficiency and stability differed. Agents with stronger attribution-violation similarity required fewer tuning steps ( 12–13 vs 22), exhibited a more concentrated TPP-tuning space with lower entropy ( 0.3 vs 0.6), converged on adjusting only a few key TPPs, and showed smaller discrepancies between practical tuning steps and the theoretical steps needed to move from initial values to the final TPP space. Putting together, these findings indicate that high-performing ACER agents can effectively identify dose violations from DVH inputs and employ a global tuning strategy to achieve high-quality treatment planning. *Significance.* This study demonstrates that the AI agent learns effective TPP-tuning strategies, exhibiting behaviors similar to those of experienced human planners. Improved interpretability of the agent’s decision-making process may enhance clinician trust and inspire new strategies for automatic treatment planning.

## 1. Introduction

Automation in radiotherapy treatment planning represents a critical component of a fully automated workflow in radiation clinics. However, several challenges exist in this field, with one of the most critical being effective and efficient automatic inverse treatment planning.

In inverse treatment planning, given clinicians’ desired clinical outcomes such as tumor coverage and organ-at-risk (OAR) sparing, the treatment planning system (TPS) optimizes machine parameters, such as beam fluence maps, to meet these objectives. This process relies heavily on the proper configuration of treatment planning parameters (TPPs), which determine how different clinical goals and constraints are weighted during optimization. Traditionally, human planners adjust these TPPs through an iterative trial-and-error process, which is both time-consuming and labor-intensive. This is particularly a challenge in time-sensitive applications, such as online adaptive radiotherapy (Li *et al* 2013, Lim-Reinders *et al* 2017). Over the past two decades, although there have been various efforts to automate this tuning process using either conventional algorithms or artificial intelligence (AI)-based strategies (Hussein *et al* 2018, Wang *et al* 2019, Fu *et al* 2021, Meyer *et al* 2021), widespread clinical adoption remains limited due to multiple challenges. Meyer *et al* (2021) reviewed multiple commercial solutions for automated planning and identified key drawbacks, including inflexibility in adapting to diverse dosimetric preferences, the requirement for large datasets to train AI models, and steep learning curves that hinder effective clinical usage. Furthermore, most existing solutions are site-specific, lacking generality across different anatomical sites or treatment techniques. AI-based solutions are typically associated with a black-box nature, leading to trust issues (Heising 2023).

To overcome these challenges, the key may lie in a deeper understanding of the inverse optimization problem itself. A better understanding of the TPP hyperspace and how individual TPP tuning contributes to trade-offs among competing dose objectives under different planning scenarios may help build generalized yet effective planning models applicable to various treatment sites and techniques. Yet, this is a complex task due to the multicriteria optimization nature of the problems (Craft *et al* 2012, Zarepisheh *et al* 2014). While such complexity can be overwhelming for human planners, it is well within the capability of AI systems (Sahiner *et al* 2019, Shan *et al* 2020, Shen *et al* 2020b). As already demonstrated in the game of Go, deep reinforcement learning (DRL)-based system AlphaGo Zero developed a global perspective on sequences of moves, enabling it to produce high-quality moves across different board positions (Silver *et al* 2017). Similarly, a DRL-based planning agent capable of learning globally optimal policies for TPP tuning could, in principle, be generalized to produce high-quality plans across diverse anatomical sites and treatment techniques.

In this regard, our groups and others have developed multiple DRL agents to operate TPSs for automatic treatment planning, achieving substantial initial progress. These DRL agents observe intermediate treatment plans generated by the TPS and automatically tune the TPPs to guide further optimization by the TPS (Shen *et al* 2019, 2020a, 2021, Liu *et al* 2022, Pu *et al* 2022, Sprouts *et al* 2022, Wang *et al* 2023, Yang *et al* 2024, Abrar *et al* 2025, Madondo e*et al* 2025). However, the reward functions used to guide DRL agent convergence during training are often tied to specific dose objectives, and the underlying decision-making process remains difficult to interpret. These limitations create a gap between the learned policies and the generality and reliability needed for broad clinical application.

To bridge this gap, we propose the use of explainable AI (EXAI) techniques (Saraswat *et al* 2022) to uncover the decision-making process of the DRL agent. In recent years, EXAI has seen growing application in cancer radiotherapy (Chatterjee *et al* 2022, Ladbury *et al* 2022, Cui *et al* 2023, Hou *et al* 2024, Teng *et al* 2024), particularly in interpreting AI models used for outcome prediction and image segmentation. For example, Hosny *et al* (2018) developed a CNN model to predict mortality risk in patients with non-small cell lung cancer from CT images. Using the EXAI technique Grad-CAM (Selvaraju *et al* 2017), they visualized the tumor and surrounding dense tissue regions as key contributors to the model’s prediction. Heising (2023) further argued that EXAI can serve as a shared mental model between clinicians and AI systems, improving collaboration and facilitating integration into clinical workflows. However, the application of EXAI in sequential decision-making tasks such as DRL-based automatic treatment planning remains unexplored.

To investigate the factors underlying the DRL agent’s effectiveness, reliability, and efficiency in automatic planning, we conducted the following study. We selected our recently developed actor-critic with experience replay (ACER)-based DRL agent (Abrar *et al* 2025) as the subject of analysis. Abrar *et al* (2025) demonstrated that training the ACER agent on a single patient case was sufficient to achieve both intra-site generalization and robustness, using prostate cancer intensity-modulated radiotherapy (IMRT) planning as the testbed. To gain further insights, we selected multiple checkpoint ACER agents from different stages of the training process and applied the integrated gradients (IGs)-based EXAI method (Sundararajan *et al* 2017) to interpret how input states contributed to the agents’ TPP-tuning decisions. We then quantified each agent’s treatment planning performance in terms of both planning quality and efficiency. To connect the input attribution analysis with planning outcomes, we further analyzed the corresponding TPP tuning space. Based on these data, we performed a systematic analysis of how ACER agents make TPP-tuning decisions that lead to effective and efficient treatment planning. We found that a well-trained ACER agent can effectively identify dose violation regions from dose-volume histogram (DVH) inputs, distinguish the planning impact of different TPP-tuning actions, and promote those actions that globally reduce the magnitude of dose violations in an effective and efficient manner. To the best of our knowledge, this study represents the first application of EXAI in automatic treatment planning.

The remainder of this paper is organized as follows. We first provide a brief overview of the ACER agent for automatic treatment planning, the IG method, and the experimental setup used for evaluating and interpreting the agent. We then present our analysis results, followed by discussion and conclusion.

## 2. Methods

### 2.1. The ACER network for automatic treatment planning

**Figure 1.**
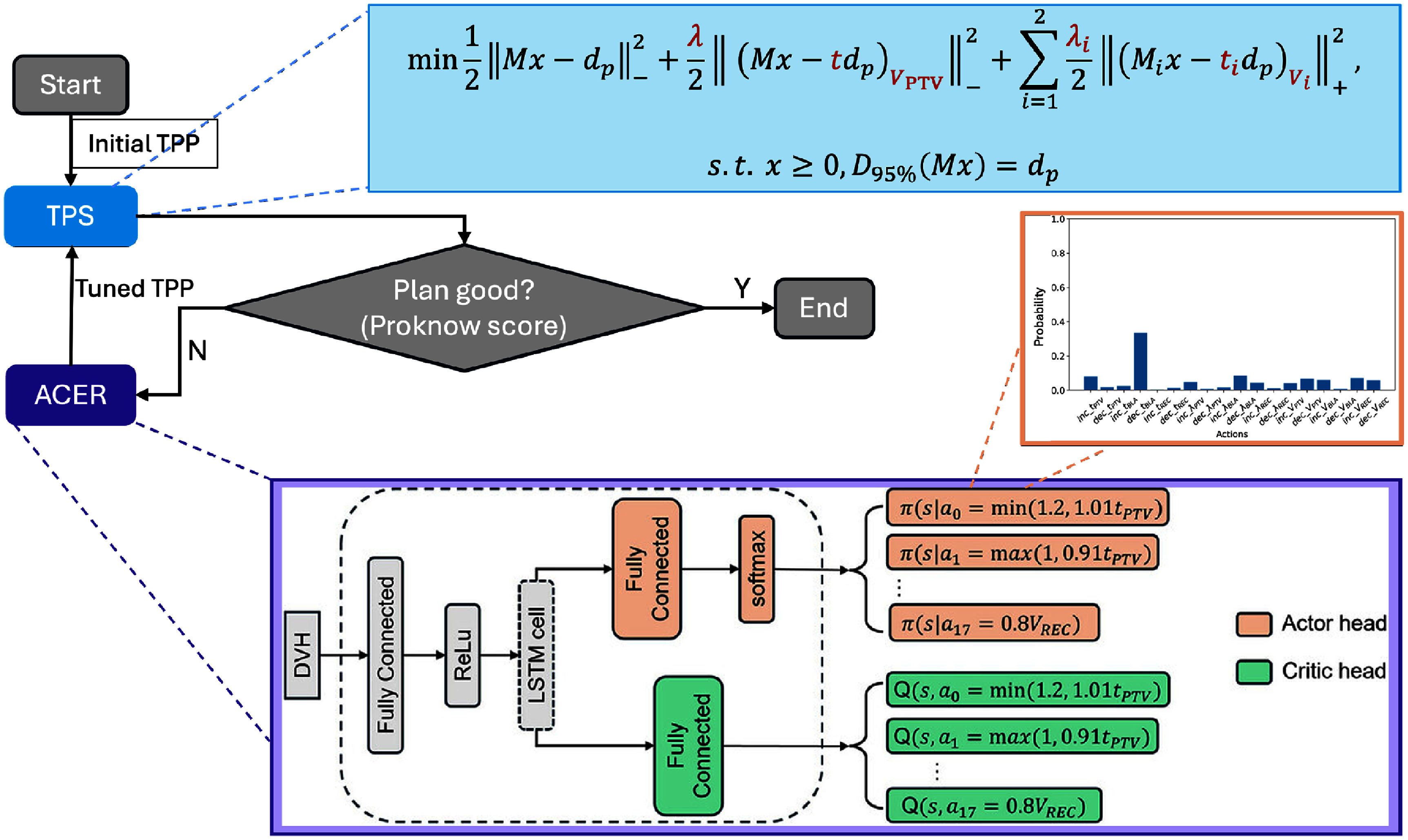
The illustration of the deep reinforcement learning (DRL) agent based automatic treatment planning for prostate cancer intensity modulated therapy.

Figure 1 illustrates the overall workflow of our ACER-based automatic treatment-planning system for prostate-cancer IMRT. In the following, we briefly describe its main components. Interested readers may refer to our previous work (Abrar *et al* 2025) for further details.

In this workflow, we employed an in-house TPS whose fluence map objective function is shown in the light black box of figure 1. There, $|\cdot|^2_-$ and $|\cdot|^2_+$ represent the under-dose and over-dose constraints, respectively. *M* denotes the dose deposition matrix, *x* is the beamlet vector, and $d_\mathrm{p}$ is the prescription dose. The adjustable TPPs include the weighting factors *λ* and *λ*_*i*_, the upper dose constraints *t* and *t_i_*, and the volume constraints $V_{\mathrm{PTV}}$ and *V_i_*. Here $i = 1, 2$ corresponds to bladder and rectum, respectively.

Starting from trivial initial TPPs, the TPS might fail to produce plans that meet clinical criteria (here, the ProKnow scoring system is employed). To address this, the ACER agent observed the DVH of the intermediate plan and generates TPP-tuning decisions. The tuned-TPPs were then fed back into the TPS for replanning. The process was iterated until a satisfying plan was obtained or the maximum number of iterations was reached.

The ACER agent itself employs a single-network, two-head architecture, with one head outputting *Q*-values and the other outputting policy distribution, as illustrated in the black box. The specific policy space constitutes 18 TPP-tuning actions, which increases or decreases each TPP value by a fixed amount. A long short-term memory (LSTM) cell is embedded in the network’s hidden layers to address training instabilities. This arises from the fact that the network receives only the DVH as user input, without access to the current TPPs or detailed evaluation metrics. As a result, the TPP tuning process forms a partially observed Markov decision process, for which LSTM has proven effective in stabilizing its training process (Hausknecht and Stone 2015, Krishnamurthy 2016, Omi *et al* 2023).

### 2.2. The IGs method

Determining the contribution of DVH inputs to ACER’s policy distributions can be framed as an attribution problem. Consider a deep network represented by a function $F: \mathbb{R}^n \rightarrow [0, 1]$ with an input $x = (x_1, \ldots, x_n) \in \mathbb{R}^n$. The attribution problem seeks to quantify the contribution of each input feature *x_i_* to the prediction *F*(*x*). This is defined relative to a baseline input *x*′, and an attribution method is supposed to assign an attribution vector $A_F(x, x^{^{\prime}}) = (a_1, \ldots, a_n) \in \mathbb{R}^n$, where *a_i_* represents the contribution of feature *x_i_* to the output.

Sundararajan *et al* (2017) proposed the IG method to obtain the attribution vector, with the IG value for the *i*th input *x_i_* to the *j*th output *F_j_* defined as

\begin{equation*} \mathrm{IG}_{ij}\left(x, x^{^{\prime}}, F_j\right) = \left(x_i - x^{^{\prime}}_i\right) \int_0^1 \frac{\partial F_j\left(x^{^{\prime}} + \alpha\left(x - x^{^{\prime}}\right)\right)}{\partial x_i} \, \mathrm{d} \alpha.\end{equation*}

Here, *F*, *x*, and *x*′ follow the definitions provided in the attribution problem, and $\alpha \in [0, 1]$ represents the interpolation coefficient along the straight path from the baseline input to the actual input.

The IG method uniquely satisfies two key axioms. The first is the sensitivity axiom, which states that input features causing different predictions should receive non-zero attributions. The second is the implementation invariance axiom, which requires that functionally equivalent networks produce identical attributions. Furthermore, the IG method satisfies the completeness property, which states that the sum of all attributions to one input, i.e. $\sum_{i = 1}^{n} \mathrm{IG}_{ij}(x, x^{^{\prime}}, F_j)$, equals the difference between $F_j(x)$ and $F_j(x^{^{\prime}})$ (Sundararajan *et al* 2017), that is

\begin{equation*} \sum_{i = 1}^{n} \mathrm{IG}_{ij}\left(x, x^{^{\prime}}, F_j\right) = F_j\left(x\right)-F_j\left(x^{^{\prime}}\right).\end{equation*}

### 2.3. Experimental setup for attribution analysis

In our application, we used the IG method to examine how DVH inputs influenced the ACER agent’s TPP-tuning decisions. While this built a connection between DVH and TPP tuning, it did not fully explain the ACER agent’s overall treatment planning efficiency and effectiveness. To complete the picture, we first examined how DVH attributions aligned with TPP-tuning strategies. We then quantified the agent’s treatment planning performance along with the associated TPP space and tuning behavior. By treating TPP tuning as a connecting bridge, we aimed to reveal how ACER effectively and efficiently performed automatic treatment planning in response to varying DVH inputs across different plans.

To support this analysis, we employed multiple checkpoint ACER agents saved at different stages of training. In our previous work (Abrar *et al* 2025), with one prostate cancer case, the ACER agent was trained for approximately 250 000 steps. The trained network showed full-score planning performance between 100 000 and 200 000 steps over two additional validation cases. A checkpoint agent at step 120 500 was used to report test performance in that study.

In the present work, to interpret the policy space and tuning behavior, we selected six checkpoint agents from training steps 100 000, 120 500, 140 000, 160 000, 180 000, and 200 000. These agents are referred to as Agent_10, Agent_C, Agent_14, Agent_16, Agent_18, and Agent_20, respectively, throughout the study. Each agent was used to perform automatic treatment planning for 39 patient cases. These cases were drawn from the same dataset and followed the same TPP initialization protocol as Test Group 1 in table 5 of our previous study (Abrar *et al* 2025), with the exception that we excluded cases that had already achieved the maximal ProKnow plan score at initialization.

#### 2.3.1. The attribution from DVH and LSTM Inputs to ACER’s TPP-tuning decisions

Before applying the IG method to our problem, it is important to revisit the LSTM cell to fully understand the structure of the ACER agent’s inputs. The specific LSTM cell used in our implementation is defined as follows:

\begin{align*} f_t &amp;= \sigma\left(W_{if} x_t + U_{hf} h_{t-1} + b_f\right), \nonumber\\ i_t &amp;= \sigma\left(W_{ii} x_t + U_{hi} h_{t-1} + b_i\right), \nonumber\\ g_t &amp;= \tanh\left(W_{ig} x_t + U_{hg} h_{t-1} + b_g\right), \nonumber\\ o_t &amp;= \sigma\left(W_{io} x_t + U_{ho} h_{t-1} + b_o\right), \nonumber\\ c_t &amp;= f_t \odot c_{t-1} + i_t \odot g_t, \nonumber\\ h_t &amp;= o_t \odot \tanh\left(c_t\right).\end{align*}

In this equation, *f_t_*, *i_t_*, *c_t_*, and *o_t_* represent the forget gate, input gate, cell state, and output gate, respectively. *h_t_* is the hidden state at time *t*. $W_{}$ and $U_{}$ denote the input and recurrent weight matrices, while *b*’s are the bias terms. The cell takes *x_t_*, the input state at time *t*, and combines it with $h_{t-1}$ and $c_{t-1}$ from the previous time step, producing *h_t_*, which is passed to the next layer and ultimately generates the policy distribution. From this structure, the ACER agent receives three inputs at each step: the user-provided *x_t_*, and the internal hidden states $h_{t-1}$ and $c_{t-1}$ from the LSTM.

Given this input configuration, we applied the IG method to quantify each input’s contribution to the agent’s policy decisions. In our application, we set the baseline values of all three inputs (DVH, *c*, and *h*) to zero. Defining the function *F* in equation (1) as the policy output for a specific TPP-tuning action, IG computed the attributions of the inputs to that action relative to the baseline. Per the completeness property of IG (Sundararajan *et al* 2017), the sum of the attributions over each input quantifies how much that input source (DVH, *c*, and *h*) increases or decreases the action’s policy probability compared to the baseline. We applied this method to compute the attribution heatmaps of the three inputs for all TPP-tuning actions over all planning steps.

To analyze the results, we conducted two complementary statistical studies:

First, we classified the attribution heatmaps into groups, each corresponding to a specific TPP-tuning action. For a given input type (DVH, *c*, or *h*), a heatmap was assigned to the group of the action with the highest total attribution. This grouping was performed separately for each input and each checkpoint agent to identify dominant attribution patterns across the policy space.

Second, we evaluated the relevance of DVH attributions by measuring their correlation with organ-wise immediate rewards. Organ-wise attribution was computed by summing the DVH input attributions for each organ. Corresponding rewards were calculated in two ways: (1) by summing the changes in Proknow scores for that organ before and after the action, and (2) by summing the changes in dose violations, defined as the volume excess of a DVH point over its corresponding Proknow score criterion. This produced a three-dimensional attribution vector and reward vector (one component per organ) for each planning step and action. We then computed the cosine similarity between the two vectors as:

\begin{equation*} \textrm{Similarity} = \frac{1}{N}\sum_{i = 1}^{N}\sum_{j = 1}^{18} p_{ij}\cos\left(\vec{A}_{ij},\vec{R}_{ij}\right).\end{equation*}

Here, *N* is the total number of TPP tuning steps, and *p*_*ij*_, $\vec{A}_{ij}$, and $\vec{R}_{ij}$ denote the probability, attribution vector, and reward vector at the *i*th planning step for the *j*th action. The normalization by *N* ensures the similarity is comparable across agents, as they may take different numbers of steps to plan the same case.

#### 2.3.2. ACER’s automatic planning performance and associated TPP tuning behavior

To evaluate each agent’s planning effectiveness and efficiency, we conducted ACER-guided automatic treatment planning for all 39 patient cases. As shown in figure 1, TPP-tuning decisions were made based on a probability distribution. To account for this stochastic nature of the decision-making process, each case was planned five times, resulting in 195 final treatment plans per agent. We then computed the means and standard deviations of the planning scores to assess effectiveness, and measured the number of planning steps per final plan to quantify efficiency. To further evaluate differences in planning efficiency among agents, statistical comparisons of the number of planning steps were performed using the linear mixed model (Seabold *et al* 2010), and the corresponding *p*-values were reported.

In the above automatic treatment planning process for the 195 plans per agent, we recorded the policy distribution at each TPP-tuning step and the final TPP configuration at the termination step. Using these records, we performed two analyses with the aim to better understand how successful agents navigate the TPP space and how their policy distributions reflect their decision strategies.

First, to identify patterns in the TPP space, we selected the treatment plans that achieved the maximal Proknow score and plotted their final TPP configurations. To quantify the convergence efficiency of each configuration, we calculated the ‘ideal’ number of planning steps, defined as the total number of monotonic, one-directional adjustments needed to reach the final TPP configuration from the initial settings.

Second, to characterize the agent’s decisiveness and strategy diversity, we calculated the entropy of the policy distribution per treatment plan as

\begin{equation*} \textrm{Entropy} = -\frac{1}{N}\sum_{i = 1}^{N}\sum_{j = 1}^{18} p_{ij}\ln\left(p_{ij}\right).\end{equation*}

Here, *p*_*ij*_ is the probability of *j*th TPP tuning action at the *i*th TPP tuning step, and *N* is the total number of TPP tuning steps. The normalization by *N* ensures the entropy is comparable across agents. Higher entropy indicates a more uniform policy distribution across actions, whereas lower entropy reflects a more concentrated distribution toward specific actions.

## 3. Results

### 3.1. The attribution from DVH and LSTM inputs to ACER’s TPP-tuning decisions

**Figure 2.**
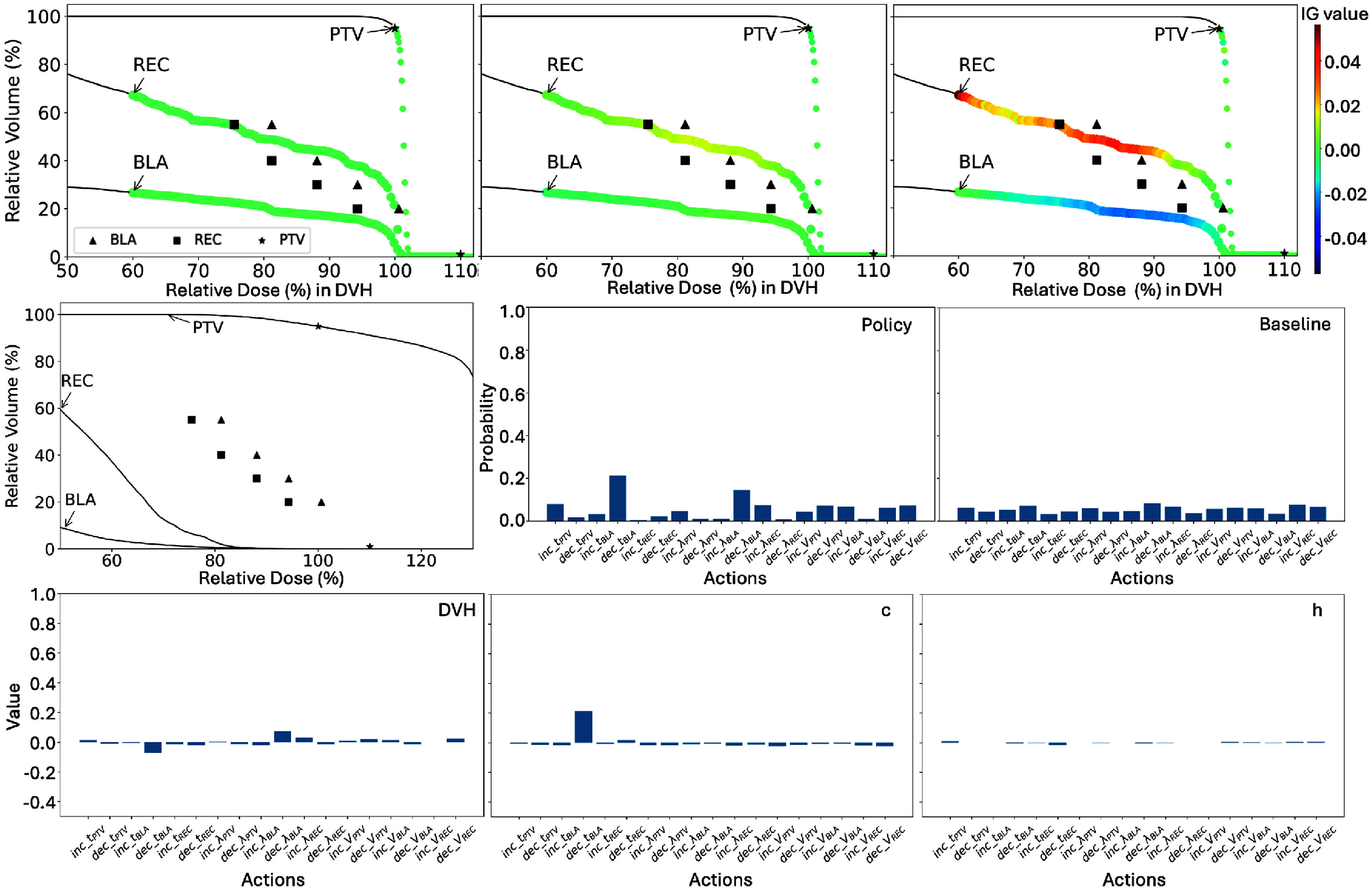
First row: DVH IG heatmaps for the leading action in a representative treatment plan from three ACER agents. DVH points were uniformly sampled along the relative dose axis for IG computation. Second and third rows: the overall policy distribution across the baseline, DVH, and memory *c* and *h* components for a representative plan and agent.

The DVH IG heatmaps contributing to a representative leading action in one TPP-tuning step for three ACER agents are shown in the first row of figure 2. In this example, the plan fails to adequately spare the rectum, as indicated by the rectum curve exceeding the corresponding Proknow evaluation criteria (square markers). The three agents are taken from training step 0, a checkpoint before Agent_10, and a checkpoint after. As training progresses, the rectum IG heatmap shifts from a neutral contribution (green) to leading action to a positive contribution (red). By the completeness property of IG, a larger total IG value corresponds to the leading action having a higher probability in the policy space. Thus, this change indicates that the agents gradually learn to detect dose violations and use this information to promote TPP-tuning actions.

The remaining two rows of figure 2 present the overall policy distribution across the baseline, DVH, and LSTM components (*c* and *h*) for a representative agent and TPP-tuning step. The action probabilities derived from the baseline input are nearly uniform, indicating that zero inputs from both DVH and LSTM produce a flat policy distribution. In contrast, the DVH and LSTM components generate distinct and non-uniform policy distributions, highlighting the agent’s sensitivity to non-zero DVH and LSTM inputs in its decision-making process.

**Figure 3.**
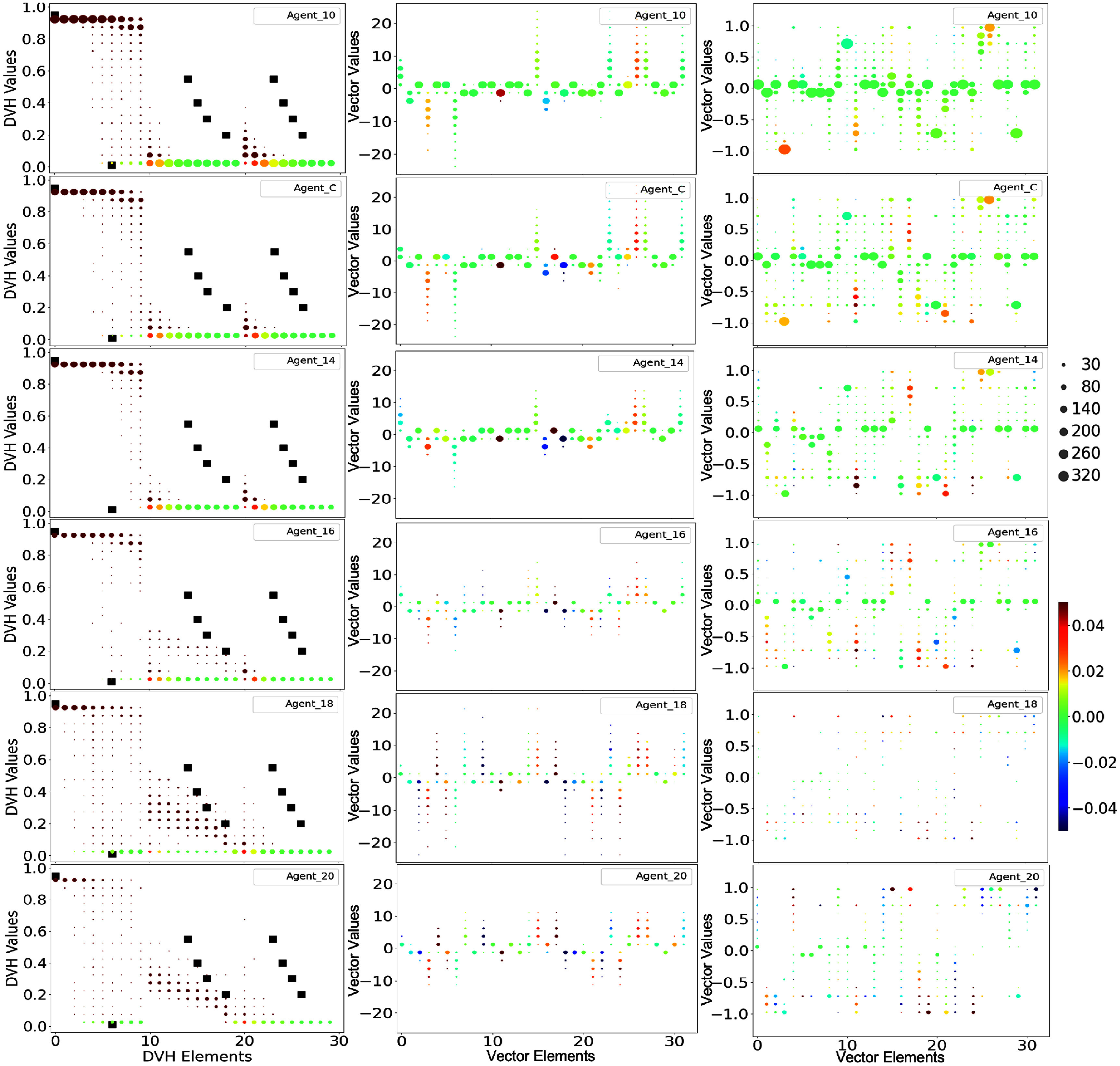
First column: attribution statistics from DVH inputs to the leading action ‘decreasing $\lambda_\mathrm{REC}$’ across all six agents. Data were collected from all planning steps of test cases where this action received the highest total IG attribution from DVH inputs. The 300 original DVH input values were grouped into 30 segments of 10 consecutive points each: indices 0–9 represent the PTV, 10–19 the bladder, and 20–29 the rectum. The ProKnow score criteria used to evaluate the plan quality are marked with black stars. Second column: corresponding attribution statistics from the LSTM memory cell state (*c*), consisting of 32 indices. Third column: attribution statistics from the LSTM hidden state (*h*), also with 32 indices.

Figure 3 shows the IG heatmap statistics from DVH and LSTM inputs to a representative leading action, ‘decreasing $\lambda_\mathrm{REC}$’. IG values for each ACER agent were collected across 78 treatment plans, corresponding to two planning runs for each of 39 patient cases. ‘decreasing $\lambda_\mathrm{REC}$’ were identified as leading action from 398, 252, 212, 128, 160, and 91 planning steps for DVH; 316, 234, 206, 76, 75, and 69 steps for cell state *c*; and 634, 452, 299, 187, 19, and 80 steps for hidden state *h*, respectively. For DVH inputs, indices 0–10, 10–20, and 20–30 correspond to the PTV, bladder, and rectum curves, respectively. The ProKnow evaluation thresholds are marked with black stars.

From the plot, PTV points typically lie above their evaluation criteria, indicating overdose, while OAR points are generally below. This implies that the agents tend to promote ‘decreasing $\lambda_\mathrm{REC}$’ action as leading action in situations with overdosed PTVs. Yet, moving from early to later agents, the level of DVH ‘hot tail’ decreases, while the bladder shows more points close to violating the plan evaluation criteria. Correspondingly, the positive IG values shift from being primarily PTV-focused to reflecting contributions from both the PTV and bladder. Since decreasing $\lambda_\mathrm{REC}$ reduces the rectum’s planning weight and effectively increases the relative importance of the PTV and bladder, later agents appear to use this action to optimize dose distribution to both structures.

**Figure 4.**
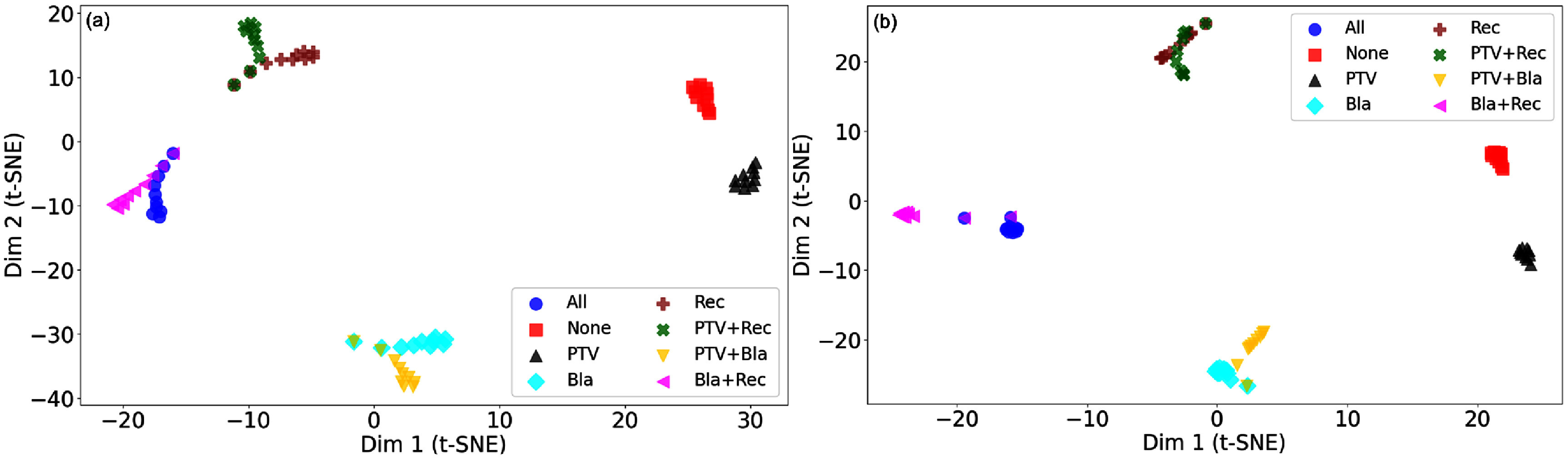
t-SNE visualization of LSTM memory representations under different dose-violation scenarios. (a) Long-term memory cell (*c*) and (b) short-term memory cell (*h*) states from the agent after processing eight representative DVH input conditions: no violation, PTV only, bladder only, rectum only, PTV + bladder, PTV + rectum, bladder + rectum, and all violations. Each point corresponds to one evolved LSTM state (10 repetitions per condition).

The LSTM attributions from the cell state *c* and hidden state *h* reveal consistent patterns. First, although *c* and *h* have quite different value ranges for each agent, the vector components with positive contributions are largely similar. Second, in early agents, most *c* values are near zero with occasional large spikes, whereas in later agents, they are more evenly distributed away from zero with fewer outliers. In parallel, the number of vector components with positive contributions increases and the observed trends align with those seen in the DVH inputs. Based on these observations, we hypothesized that LSTM states act as dose violation memory. To test it, we performed a t-SNE analysis for both long-term memory cell *c* and short-term memory cell *h*. Specifically, two input scenarios were defined: a DVH curve of all ones (‘violation’) and all zeros (‘conserved’). Using these, we constructed eight cases: no violation, PTV only, bladder only, rectum only, PTV + bladder, PTV + rectum, bladder + rectum, and all violations. For each case, the agent processed the same DVH input ten times to evolve its LSTM memory. The resulting 80 *c* vectors and 80 *h* vectors were normalized and analyzed using t-SNE (perplexity = 8, cosine metric, PCA initialization, learning rate = 50). As shown in figure 4, distinct clusters corresponding to different violation regions were observed for both *c* and *h* cells, supporting the hypothesis that LSTM states serve as dose-violation memory.

**Figure 5.**
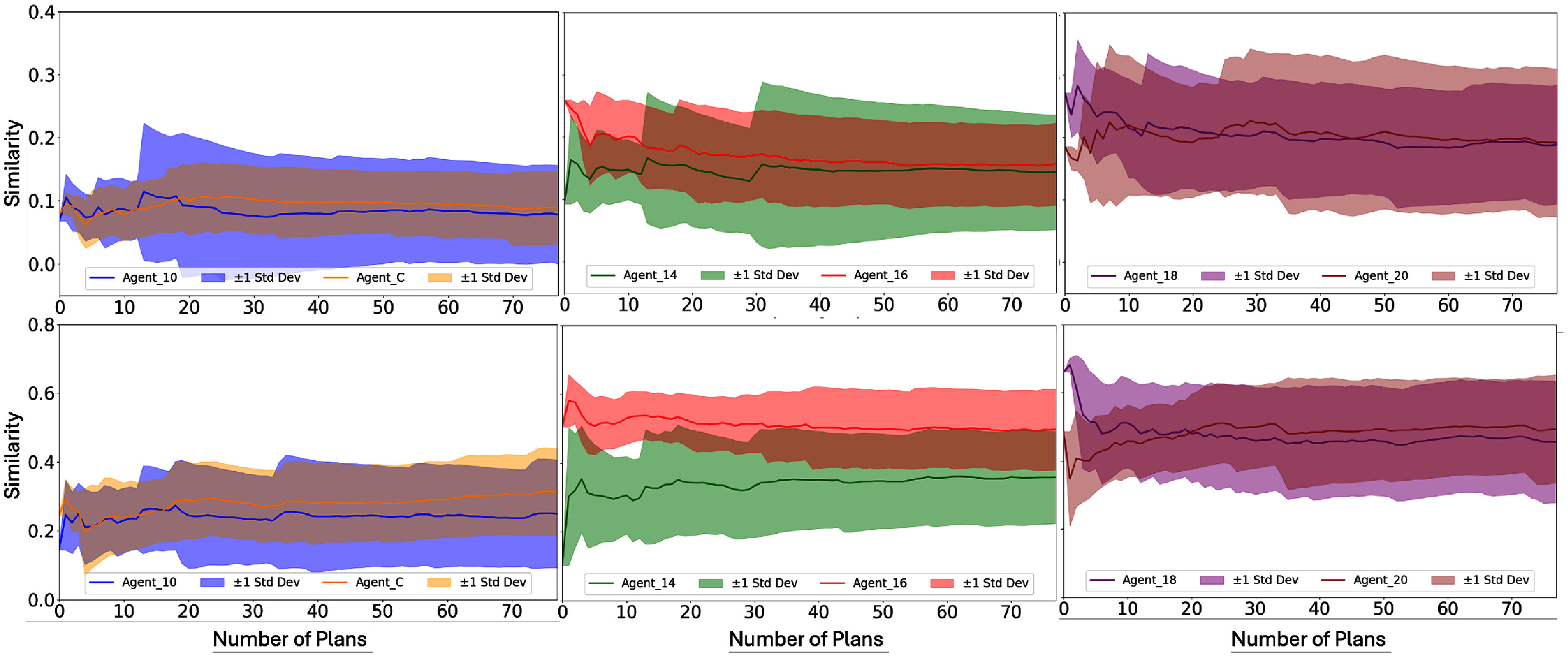
Similarity between organ-wise attribution and organ-wise immediate reward across the six ACER agents. The top row shows results using reward calculation Method 1 (score difference), while the bottom row uses Method 2 (dose violation difference).

Figure 5 gives the similarity between organ-wise attribution and organ-wise immediate reward for the six ACER agents. In the top row (Reward 1: score difference), Agents 18 and 20 exhibit the highest similarity (around 0.2), followed by Agents 14 and 16 (around 0.15), while Agents 10 and C remain below 0.1. In the bottom row (Reward 2: dose violation difference), all agents demonstrate much higher similarity values. Agents 16, 18, and 20 show the strongest alignment (around 0.5), with a noticeable gap from the remaining agents. Defining 0.15 as ‘high’ similarity under Reward 1 and 0.4 under Reward 2, we observe that later-stage agents (Agents 16, 18, and 20) consistently achieve high similarity levels under both reward definitions. Taking Agent_16 as the reference, we performed repeated-measures ANOVA to assess statistical significance in similarity differences across agents. Under Reward 1, Agents 10 and C exhibited significantly lower similarity than Agent 16 ($p < $ 0.01), while directional *p*-values for Agents 14, 18, and 20 were −0.32, +0.074, and +0.096, respectively, indicating no significant difference. Here, the sign denotes whether the mean similarity is lower (−) or higher (+) than that of Agent_16. Under Reward 2, Agents 10, C, and 14 showed significantly lower similarity than Agent_16 ($p < $ 0.01), whereas directional *p*-values for Agents 18 and 20 were −0.085 and +0.85, respectively, again indicating no significant difference. The consistently higher and more distinguishable similarity under Reward 2 suggests that it better reflects the agents’ decision-making process. In particular, once an agent identifies an organ dose violation, the action it promotes within that TPP-tuning step may not immediately improve the plan score, but it can reduce the magnitude of that dose violation. With a well-aligned dose-violation observation and an action to mitigate it, the agent can, in principle, effectively improve the plan quality to ultimately achieve the highest score.

### 3.2. ACER’s automatic planning performance and associated TPP tuning behavior

Table 1 presents statistical summaries of the planning effectiveness and efficiency for each ACER agent. From the table, all agents improve the plan qualities from a Proknow score of $5.15\pm1.72$ before planning to nearly the maximum of 9 after planning. However, there is a large variation among agents in the number of plans reaching the maximum Proknow score of 9, ranging from 143 for Agent_10 to 191 for Agent_16 out of 195 planning cases. The number of planning steps to get these full-score plans also varies substantially: Agent_10 requires about $22.4\pm5.5$ steps for planning, while later agents, such as Agent_18 generate full-score plans in nearly half the steps ($12.19\pm3.57$). Particularly, Agent_16 achieves the highest plan score with the second-fastest planning speed, both with the smallest standard deviations, demonstrating stable, high performance. The *p*-value results show that efficiency of Agent_16 differs significantly from all other agents except for Agent_20.

**Table 1.** Treatment planning performance comparison among the six ACER agents. The initial and final plan scores were computed over 195 planning cases. The number of treatment plans (out of the 195) that achieved the maximum Proknow score of 9 is reported as ‘# of 9’s’. The required number of planning steps and the ‘ideal’ number of planning steps were calculated from this subset of plans. *p*-values indicate the statistical significance of the differences in (ideal) number of planning steps compared with Agent_16.

Agent	Initial score	Final score	# of 9’s	# of steps	*p*	Ideal # of steps	*p*
Agent_10	$5.75 \pm 1.72$	$8.75 \pm 0.44$	143	$22.4 \pm 5.5$	$ < $.01	$15.0 \pm 3.1$	$ < $.01
Agent_C		$8.90 \pm 0.32$	176	$18.0 \pm 4.2$	$ < $.01	$13.0 \pm 2.1$	$ < $.01
Agent_14		$8.98 \pm 0.16$	190	$16.0 \pm 2.9$	$ < $.01	$13.0 \pm 1.7$	$ < $.01
Agent_16		$8.99 \pm 0.08$	191	$13.7 \pm 2.5$	—	$11.1 \pm 1.6$	—
Agent_18		$8.93 \pm 0.30$	177	$12.2 \pm 3.6$	$ < $.01	$9.4 \pm 2.1$	$ < $.01
Agent_20		$8.98 \pm 0.16$	185	$14.0 \pm 5.1$	0.44	$9.1 \pm 1.8$	$ < $.01

**Figure 6.**
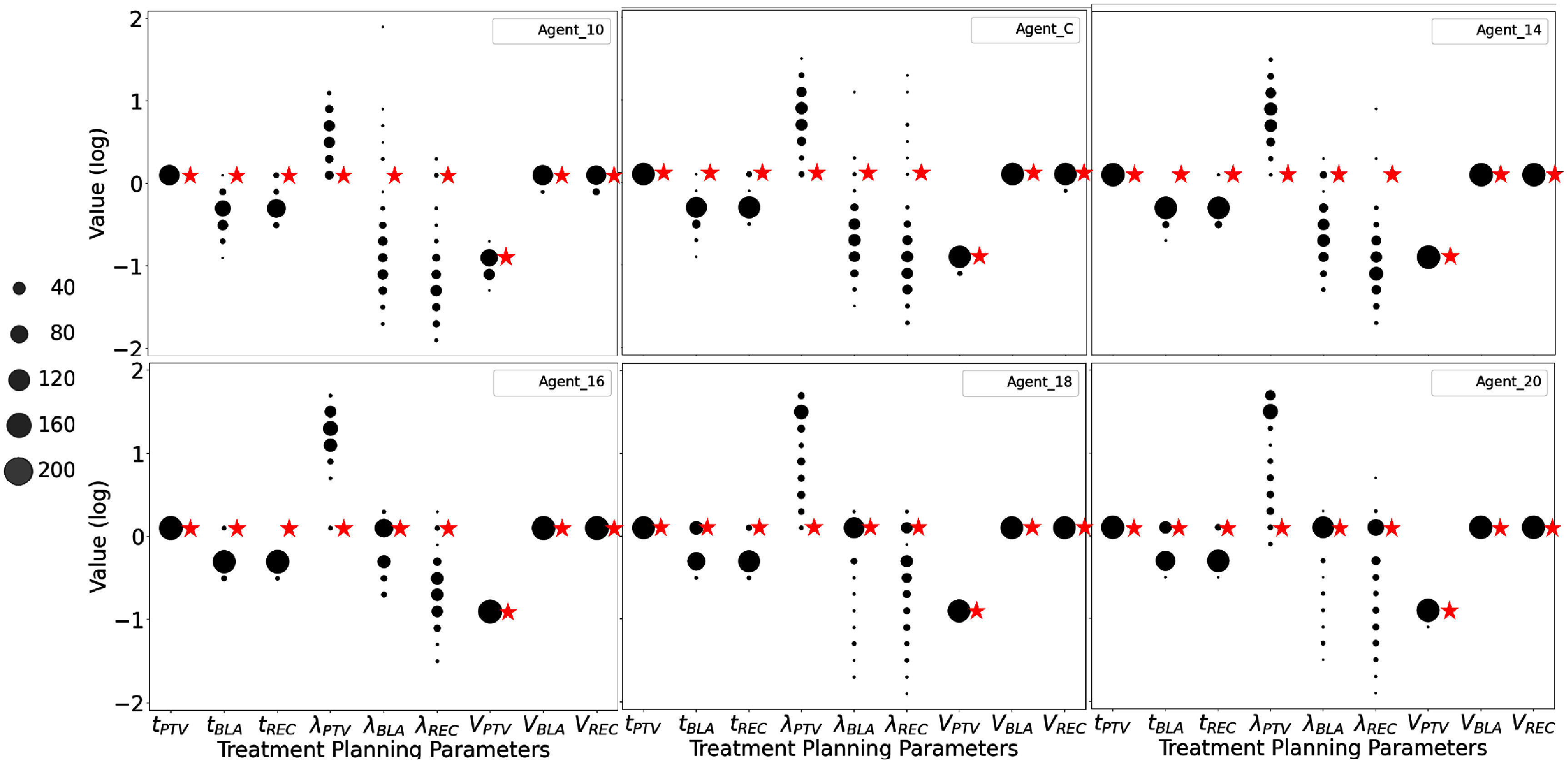
The value distributions in the treatment planning parameter (TPP) space corresponding to planning cases that achieved the maximum score of 9 for each of the six ACER agents. The red starred points indicate the initial TPP values prior to treatment planning, while the dotted points represent the final TPP values. The size of each dot is proportional to the number of planning cases that converged to that specific TPP value.

Figure 6 shows the final TPP space for plans that achieve a final planning score of 9. The six agents exhibit similar distribution patterns over the final TPP values. For example, the main tuning focus is on the weighting parameters *λ*’s, indicating shared tuning priorities. However, differences still exist. Comparing to other agents, Agent_10’s dose thresholds $t_\mathrm{REC}$ and $t_\mathrm{BLA}$ converge to more distinct values. Both Agent_10 and Agent_C display bidirectional convergence in $\lambda_\mathrm{REC}$ and $\lambda_\mathrm{BLA}$, while the rest tend to converge in only one direction. Later agents converge to fewer distinct TPP points (e.g. the fewest dots in Agent_16) and preserve more of their initial TPP settings, as shown by the larger black dots near the red-star markers (e.g. for $\lambda_\mathrm{REC}$ in Agent_16, Agent_18 and Agent_20).

The ‘ideal’ number of planning steps is then listed in the last column of table 1, which decreases from around 15 in Agent_10 to about 9 in Agent_20. This trend is consistent with the final TPP space distribution patterns that later agents retain more initial TPP values than early agents. However, the actual planning steps (second-to-last column) remain higher than these values, reflecting back-and-forth TPP tunings during the planning process. Nonetheless, later agents exhibit a smaller discrepancy between the two measures, with Agent_16 reaching a minimum.

**Figure 7.**
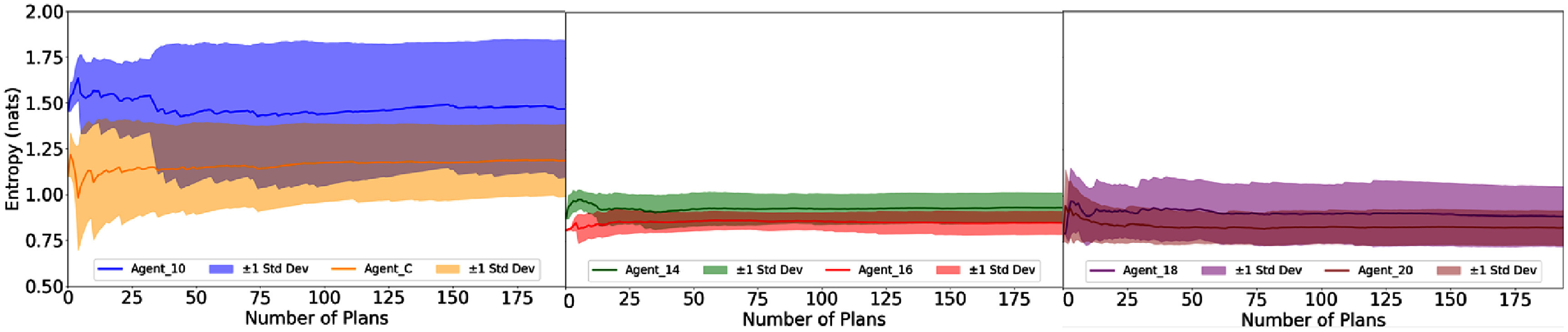
Entropy (in ln unit) of the policy space plotted against the number of treatment plans for the six ACER agents.

The entropy of the policy space is illustrated in figure 7. Later agents display lower entropy with smaller standard deviation compared to earlier agents (e.g. Agent_10, Agent_C). Since lower entropy corresponds to more non-uniform distributions, this suggests that later agents have more dominant leading actions in the policy space.

### 3.3. Overall interpretation of ACER-based automatic planning

In this subsection, we connect the results from subsection 3.1 with those from subsection 3.2 to provide an overall interpretation of the decision-making process in ACER-based automatic treatment planning.

From table 1, later agents such as Agent_16 demonstrate superior performance in both planning quality and efficiency compared with earlier agents such as Agent_10 and Agent_C. The following paragraphs present our interpretation of this behavior.

**Figure 8.**
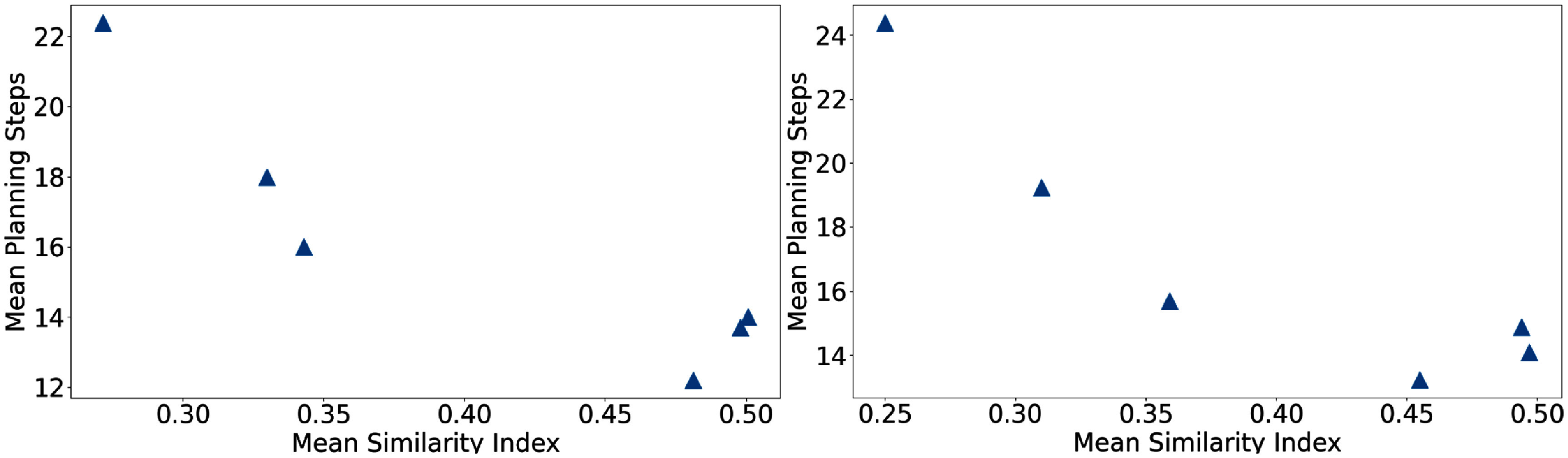
Correlation between similarity (attribution to plan improvement) under Reward 2 (dose violation difference) and mean planning steps. Left: treatment plans achieving a full plan score of 9. Right: all test cases summarized in table 1.

From figures 2 and 3, later agents show better recognition of actual and potential dose violations and display more positive IG distributions toward leading actions, thereby increasing the probability of these actions in the policy space. This is consistent with the entropy analysis in figure 7, which shows that later agents have more dominating leading actions. From figure 5, these agents achieve high similarities between organ-wise attribution and dose violation mitigation, indicating the effectiveness of these promoted actions. We further correlated this similarity behavior with corresponding planning steps listed in table 1 and showed the result in figure 8. Clearly, a strong negative correlation exists between the mean similarity index and the mean number of planning steps, observed both across all test cases and within full-score plans. This indicates that agents exhibiting higher similarity values also converge more efficiently. This can be understood that the promoted actions have a global effect, systematically improving plan quality without back-and-forth adjustments of a single TPP parameter. After several TPP-tuning steps, this naturally produces high-quality treatment plans. The uncertainty levels in the similarity and entropy analyses also align well with those for plan quality and planning steps in table 1, further supporting this reasoning.

Meanwhile, although later agents all show high effectiveness in achieving high final planning scores, their planning speeds and the number of plans that reach the maximum Proknow score vary. The variation can be interpreted as follows. The final TPP space distribution (figure 6) shows that the promoted actions are primarily ‘increasing $\lambda_\mathrm{PTV}$’, ‘reducing $\lambda_\mathrm{REC}$’, and ‘reducing $\lambda_\mathrm{BLA}$’. Because only the relative values of the weighting parameters *λ*’s matter in the inverse optimization problem(figure 1), increasing $\lambda_\mathrm{PTV}$ is effectively equivalent to reducing both $\lambda_\mathrm{REC}$ and $\lambda_\mathrm{BLA}$. Agents 14 to 20 all mainly focus on tuning these *λ* values, thus achieving similarly high-quality treatment planning. However, as training progresses from Agent_14 to Agent_20, the focus shifts toward solely increasing $\lambda_\mathrm{PTV}$, which shortens tuning steps and improves efficiency. In some situations, though, individually tuning these *λ*’s are important. This strategy is used by Agent_16, which achieves the highest planning effectiveness and the second-fastest planning speed. By contrast, later agents such as Agent_18 and Agent_20 rely too heavily on solely increasing $\lambda_\mathrm{PTV}$. This approach increases their planning speed but reduces flexibility, resulting in fewer maximum-score plans compared to Agent_16.

## 4. Discussion

In this study, we examined the attributions underlying the ACER agent’s intelligent performance in automatic treatment planning. We found that an ACER agent with effective and efficient planning behavior can identify dose violations from DVH inputs and promote proper TPP-tuning actions to reduce the violation magnitude. After several tuning steps, these actions result in the generation of high-quality treatment plans. From a training perspective, ProKnow evaluation criteria were integrated into ACER’s immediate reward and thus the accumulated reward. To maximize this reward, the agent needed to separate individual dose-violation signals from the overall feedback and identify which TPP-tuning actions can most effectively mitigate these violations, as future rewards were discounted. This learned understanding helps explain why, as shown in our previous work (Abrar *et al* 2025), an ACER agent trained on a single prostate IMRT patient case can achieve high-quality treatment planning across prostate IMRT cases with varying anatomical overlaps between PTV and OARs and different beam configurations.

Although the treatment planning scenario used in this study was relatively simple, involving only one PTV and two OARs, the conclusions drawn from it can still offer meaningful insights into practical treatment planning by clinical TPSs. From a naive perspective, it can be difficult to distinguish the effects of tuning different TPPs on improving plan quality. Yet, the ACER agent learns to differentiate their relative importance through training. As shown in figure 6, the agent discovers that by primarily adjusting the *λ* parameters, plan quality can be improved both effectively and efficiently. In figure 3, when dose violations appear in both the PTV and one OAR, the agent chooses to reduce the *λ* for the other OAR. Under Reward 2 definition (dose violation difference), we specifically evaluated how this action affects dose coverage for both PTV and the involved OAR. We found that in 77.7%, 79.8%, and 76.0% of such tuning instances for Agents 16, 18, and 20, respectively, the adjustment helps mitigate violations in both regions. This indicates that such actions have a global tuning effect and can thus be regarded as high-quality TPP-tuning strategies. Interestingly, in clinical practice, experienced human planners also tend to tune only a few key parameters with minimal tuning steps. The AI agent appears to acquire similar experience during training, much like a human.

Building on the above insights, several key directions can be pursued to extend this work toward practical clinical applications and broader generality. A first step is to extend the ACER framework to handle more complex PTV and OAR configurations by enlarging its DVH input layer and output branches, while incorporating dose-evaluation criteria for additional structures into the reward function. Owing to the framework’s scalability, such extensions are technically feasible. Another important direction is refining the reward function to enhance clinical robustness. Our analysis suggests that the ACER agent can identify dose-violation criteria embedded in its reward design and use this information to guide TPP tuning. In clinical practice, however, dose-evaluation standards vary across institutions and tumor sites. To ensure robust performance under such variability, the reward function should promote deeper exploration among competing objectives, potentially imposing stricter constraints than those used in current clinical protocols. Finally, since the learned behavior of ACER is inherently reward-dependent, a model trained under a single reward definition may not generalize to tumor sites governed by different evaluation criteria. A promising strategy is to train a unified network under multiple representative reward functions, analogous to how a general DRL agent can master different games such as chess, shogi, and Go simultaneously (Silver *et al* 2018). Through these continued developments, we expect to build a DRL agent that achieves generality and reliability in real-world clinical applications. In addition, the acquired TPP tuning knowledge could also assist the building of other AI models for effective planning.

Meanwhile, as demonstrated in the game of Go, the DRL-based AlphaGo Zero agent not only defeated top human players but also played an educational role by helping human players develop novel moves in challenging games (Shin *et al* 2023). Similarly, by quantitatively unraveling the black box of TPP-tuning effectiveness in complex inverse treatment planning, this knowledge can be used for educational purposes to train human planners. This understanding could further facilitate the development of a more interactive human–AI environment. For instance, by developing a user interface that allows clinicians to visualize IG heatmaps during planning, human planners could intervene when an unreasonable AI decision is detected. Conversely, if clinicians aim to particularly restrict the dose to a specific OAR, heatmap distributions can help identify those most effective actions, enabling planners to effectively achieve the desired planning goal.

**Figure 9.**
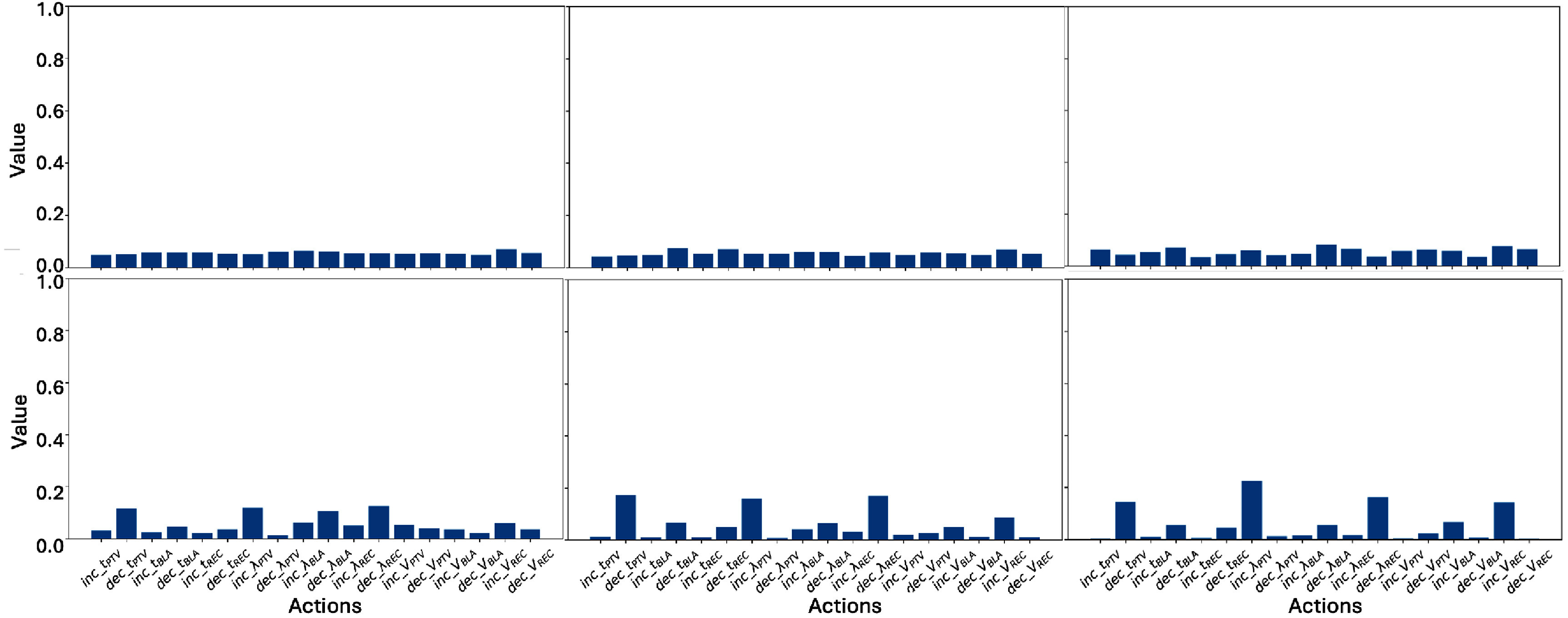
DVH backgrounds for a same DVH input obtained under zero baseline input for ACER agents at training steps 0, 40 000, 80 000, 120 000, 160 000, and 200 000.

**Figure 10.**
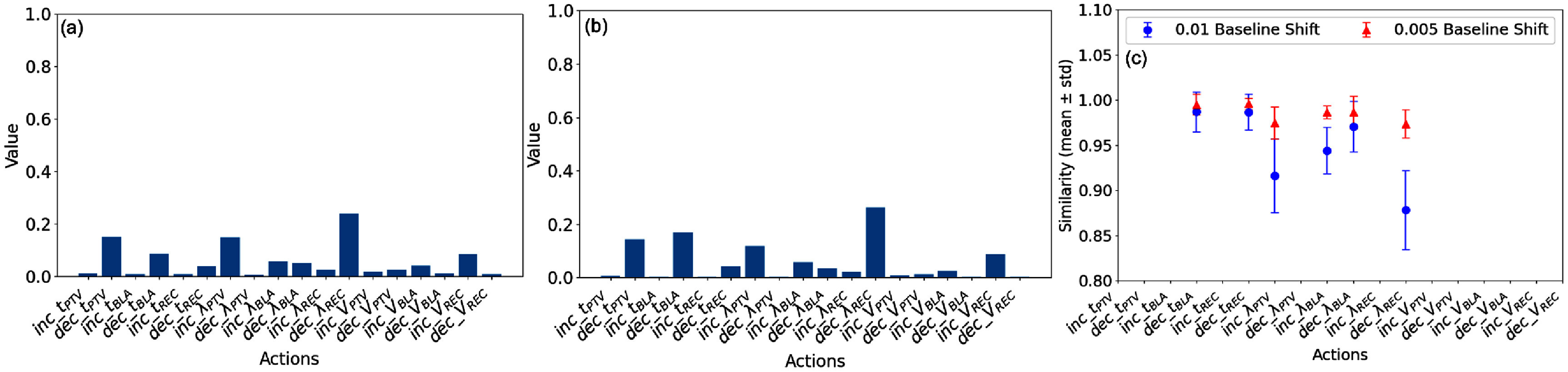
DVH backgrounds obtained under baseline offsets of (a) 0.005 and (b) 0.1 for ACER agent at training step 160 000. (c) Mean and standard deviation for cosine similarities between DVH heatmaps obtained under the shifted baselines and those obtained under the original zero baseline across all 39 patient cases for the leading action at each planning step.

Except for the discussion of clinical impact, we would also like to address some technical considerations in the current EXAI study and outline a plan for future improvements. In this study, we used zero inputs as the baseline, which offered a convenient and uniform background at the start of training. However, as training progresses, later agents encountered different sets of treatment plans compared to earlier agents, resulting in a biased background when using zero inputs, as illustrated in figure 9. This violates the assumption of baseline neutrality and may influence the quantitative values in the DVH attribution analysis. Despite this, we argue that the use of a zero-input baseline represents a complete absence of planning signal and thus enables a fair comparison of attribution evolution throughout training. Yet, we also wanted to quantify its impact on DVH attribution analysis and thus conducted a sensitivity analysis as follows. Specifically, we chose Agent_16 and offset the zero DVH baseline to 0.005 and 0.01, except for PTV points, which were kept at zero to avoid violating ProKnow dose criteria. We found that the resulting background distributions differed substantially from those generated under the zero baseline as shown in figures 10(a) and (b). We then computed the cosine similarity between DVH heatmaps obtained using the offset baselines and those from the original zero baseline across all planning steps of the 39 patient cases for the leading action at each step. The resulting similarities were grouped by action, forming six categories: ‘decrease $t_{\mathrm{BLA}}$’, ‘decrease $t_{\mathrm{REC}}$’, ‘increase $\lambda_{\mathrm{PTV}}$’, ‘increase $\lambda_{\mathrm{BLA}}$’, ‘decrease $\lambda_{\mathrm{BLA}}$’, and ‘decrease $\lambda_{\mathrm{REC}}$’, containing 48, 68, 331, 4, 25, and 130 values, respectively. Finally, we computed the mean and standard deviation for each action group and plotted them in figure 10(c). As shown, all heatmaps exhibited high similarity, exceeding $97.4 \pm 1.6\%$ under the baseline offset of 0.005 and $87.8 \pm 4.3\%$ under the 0.01 offset. These results confirm that the DVH attribution trends remain robust to baseline bias. Nonetheless, to achieve a more uniform and neutral background across agents, dynamic baseline inputs may be needed, following methods such as those described in Morasso *et al* (2025). An unbiased baseline would not only enable more precise input attribution but may also reveal inherent properties of the trained agents, contributing to a deeper understanding of their behavior. We plan to investigate this direction in future work.

## 5. Conclusion

In this study, we performed attribution analysis to understand the decision-making process in ACER-based automatic treatment planning. We found that well-trained ACER agents can effectively identify dose violation regions on DVH inputs and promote appropriate TPP-tuning actions to address these violations. This leads to the discovery of efficient TPP-tuning paths to achieve full-score plans, thereby achieving high efficacy and efficiency in treatment planning.

## Data Availability

The data cannot be made publicly available upon publication because no suitable repository exists for hosting data in this field of study. The data that support the findings of this study are available upon reasonable request from the authors.

## References

[pmbae2561bib1] Abrar M M, Sapkota P, Sprouts D, Jia X, Chi Y (2025). Actor critic with experience replay-based automatic treatment planning for prostate cancer intensity modulated radiotherapy. https://arxiv.org/abs/2502.00346.

[pmbae2561bib2] Chatterjee S, Das A, Mandal C, Mukhopadhyay B, Vipinraj M, Shukla A, Nagaraja Rao R, Sarasaen C, Speck O, Nürnberger A (2022). Torchesegeta: framework for interpretability and explainability of image-based deep learning models. Appl. Sci..

[pmbae2561bib3] Craft D L, Hong T S, Shih H A, Bortfeld T R (2012). Improved planning time and plan quality through multicriteria optimization for intensity-modulated radiotherapy. Int. J. Radiat. Oncol. Biol. Phys..

[pmbae2561bib4] Cui S, Traverso A, Niraula D, Zou J, Luo Y, Owen D, El Naqa I, Wei L (2023). Interpretable artificial intelligence in radiology and radiation oncology. Br. J. Radiol..

[pmbae2561bib5] Fu Y (2021). Artificial intelligence in radiation therapy. IEEE Trans. Radiat. Plasma Med. Sci..

[pmbae2561bib6] Hausknecht M J, Stone P (2015). Deep recurrent Q-learning for partially observable MDPs. AAAI Fall Symposia.

[pmbae2561bib7] Heising L (2023). Accelerating implementation of artificial intelligence in radiotherapy through explainability.

[pmbae2561bib8] Hosny A (2018). Deep learning for lung cancer prognostication: a retrospective multi-cohort radiomics study. PLoS Med..

[pmbae2561bib9] Hou J, Liu S, Bie Y, Wang H, Tan A, Luo L, Chen H (2024). Self-explainable AI for medical image analysis: a survey and new outlooks. https://arxiv.org/abs/2410.02331.

[pmbae2561bib10] Hussein M, Heijmen B J M, Verellen D, Nisbet A (2018). Automation in intensity modulated radiotherapy treatment planning-a review of recent innovations. Br. J. Radiol..

[pmbae2561bib11] Krishnamurthy V (2016). Partially Observed Markov Decision Processes.

[pmbae2561bib12] Ladbury C, Zarinshenas R, Semwal H, Tam A, Vaidehi N, Rodin A S, Liu A, Glaser S, Salgia R, Amini A (2022). Utilization of model-agnostic explainable artificial intelligence frameworks in oncology: a narrative review. Transl. Cancer Res..

[pmbae2561bib13] Li N (2013). Automatic treatment plan re-optimization for adaptive radiotherapy guided with the initial plan DVHS. Phys. Med. Biol..

[pmbae2561bib14] Lim-Reinders S, Keller B M, Al-Ward S, Sahgal A, Kim A (2017). Online adaptive radiation therapy. Int. J. Radiat. Oncol. Biol. Phys..

[pmbae2561bib15] Liu Y, Shen C, Wang T, Zhang J, Yang X, Liu T, Kahn S, Shu H-K, Tian Z (2022). Automatic inverse treatment planning of gamma knife radiosurgery via deep reinforcement learning. Med. Phys..

[pmbae2561bib16] Madondo M, Shao Y, Liu Y, Zhou J, Yang X, Tian Z (2025). Patient-specific deep reinforcement learning for automatic replanning in head-and-neck cancer proton therapy. https://arxiv.org/abs/2506.10073.

[pmbae2561bib17] Meyer P, Biston M-C, Khamphan C, Marghani T, Mazurier J, Bodez V, Fezzani L, Rigaud P, Sidorski G, Simon L (2021). Automation in radiotherapy treatment planning: examples of use in clinical practice and future trends for a complete automated workflow. Cancer/Radiothérapie.

[pmbae2561bib18] Morasso C, Dolci G, Galazzo I B, Plis S M, Menegaz G (2025). Guidelines for the choice of the baseline in xai attribution methods. https://arxiv.org/abs/2503.19813.

[pmbae2561bib19] Omi S, Shin H-S, Cho N, Tsourdos A (2023). Dynamic deep-reinforcement-learning algorithm in partially observed markov decision processes. https://arxiv.org/abs/2307.15931.

[pmbae2561bib20] Pu G, Jiang S, Yang Z, Hu Y, Liu Z (2022). Deep reinforcement learning for treatment planning in high-dose-rate cervical brachytherapy. Phys. Medica.

[pmbae2561bib21] Sahiner B, Pezeshk A, Hadjiiski L M, Wang X, Drukker K, Cha K H, Summers R M, Giger M L (2019). Deep learning in medical imaging and radiation therapy. Med. Phys..

[pmbae2561bib22] Saraswat D, Bhattacharya P, Verma A, Prasad V K, Tanwar S, Sharma G, Bokoro P N, Sharma R (2022). Explainable AI for healthcare 5.0: opportunities and challenges. IEEE Access.

[pmbae2561bib23] Seabold S, Perktold J (2010). Statsmodels: econometric and statistical modeling with python. SciPy.

[pmbae2561bib24] Selvaraju R R, Cogswell M, Das A, Vedantam R, Parikh D, Batra D (2017). Grad-CAM: visual explanations from deep networks via gradient-based localization.

[pmbae2561bib25] Shan H, Jia X, Yan P, Li Y, Paganetti H, Wang G (2020). Synergizing medical imaging and radiotherapy with deep learning. Mach. Learn.: Sci. Technol..

[pmbae2561bib26] Shen C, Chen L, Jia X (2021). A hierarchical deep reinforcement learning framework for intelligent automatic treatment planning of prostate cancer intensity modulated radiation therapy. Phys. Med. Biol..

[pmbae2561bib27] Shen C, Gonzalez Y, Klages P, Qin N, Jung H, Chen L, Nguyen D, Jiang S B, Jia X (2019). Intelligent inverse treatment planning via deep reinforcement learning, a proof-of-principle study in high dose-rate brachytherapy for cervical cancer. Phys. Med. Biol..

[pmbae2561bib28] Shen C, Nguyen D, Chen L, Gonzalez Y, McBeth R, Qin N, Jiang S B, Jia X (2020a). Operating a treatment planning system using a deep-reinforcement learning-based virtual treatment planner for prostate cancer intensity-modulated radiation therapy treatment planning. Med. Phys..

[pmbae2561bib29] Shen C, Nguyen D, Zhou Z, Jiang S B, Dong B, Jia X (2020b). An introduction to deep learning in medical physics: advantages, potential and challenges. Phys. Med. Biol..

[pmbae2561bib30] Shin M, Kim J, Van Opheusden B, Griffiths T L (2023). Superhuman artificial intelligence can improve human decision-making by increasing novelty.

[pmbae2561bib31] Silver D (2017). Mastering the game of go without human knowledge. Nature.

[pmbae2561bib32] Silver D (2018). A general reinforcement learning algorithm that masters chess, shogi and Go through self-play. Science.

[pmbae2561bib33] Sprouts D, Gao Y, Wang C, Jia X, Shen C, Chi Y (2022). The development of a deep reinforcement learning network for dose-volume-constrained treatment planning in prostate cancer intensity modulated radiotherapy. Biomed. Phys. Eng. Express.

[pmbae2561bib34] Sundararajan M, Taly A, Yan Q (2017). Axiomatic attribution for deep networks.

[pmbae2561bib35] Teng Z (2024). A literature review of artificial intelligence (AI) for medical image segmentation: from AI and explainable AI to trustworthy ai. Quant. Imaging Med. Surg..

[pmbae2561bib36] Wang C, Zhu X, Hong J C, Zheng D (2019). Artificial intelligence in radiotherapy treatment planning: present and future. Technol. Cancer Res. Treat..

[pmbae2561bib37] Wang H, Bai X, Wang Y, Lu Y, Wang B (2023). An integrated solution of deep reinforcement learning for automatic IMRT treatment planning in non-small-cell lung cancer. Front. Oncol..

[pmbae2561bib38] Yang D, Wu X, Li X, Mansfield R, Xie Y, Wu Q, Wu Q J, Sheng Y (2024). Automated treatment planning with deep reinforcement learning for head-and-neck (HN) cancer intensity modulated radiation therapy (IMRT). Phys. Med. Biol..

[pmbae2561bib39] Zarepisheh M, Uribe-Sanchez A F, Li N, Jia X, Jiang S B (2014). A multicriteria framework with voxel-dependent parameters for radiotherapy treatment plan optimization. Med. Phys..

